# Draft Genome Sequence of *Streptomyces* sp. Strain C8S0, Isolated from a Highly Oligotrophic Sediment

**DOI:** 10.1128/MRA.01441-19

**Published:** 2020-04-02

**Authors:** S. Gallegos-Lopez, P. M. Mejia-Ponce, L. A. Gonzalez-Salazar, L. Rodriguez-Orduña, V. Souza-Saldivar, C. Licona-Cassani

**Affiliations:** a Centro de Biotecnología-FEMSA, Tecnológico de Monterrey, Monterrey, Mexico; b Departamento de Ecología Evolutiva, Instituto de Ecología, Universidad Nacional Autónoma de México, Coyoacán, Mexico; University of Southern California

## Abstract

*Streptomyces* spp. are prolific bacteria producing bioactive metabolites. We present the draft genome sequence of *Streptomyces* sp. strain C8S0, which was isolated from a highly oligotrophic sediment from the Cuatro Cienegas Basin (Mexico). The whole-genome assembly comprised 6,898,902 bp, with 18 biosynthetic gene clusters, including those for nonconventional terpenes, nonribosomal peptides, and polyketides.

## ANNOUNCEMENT

The Cuatro Cienegas Basin is famous for harboring morphologically diverse stromatolites and microbial mats that resemble the conditions of ancient oceans (1, 2). Biogeochemical analyses have shown a markedly unbalanced nitrogen-to-phosphorus stoichiometry, mostly attributable to the extremely low phosphorus availability (3). These unusual environmental conditions are the main driving force shaping unique metabolic traits of diverse microbial communities (4). In particular, *Streptomyces* species have been proposed as prolific natural product producers due to their diverse specialized metabolisms. In order to explore the entire biosynthetic potential and to provide insights into metabolic mechanisms of adaptation, we isolated and sequenced the genome of *Streptomyces* sp. strain C8S0.

We collected soil samples from the Churince hydrological system (26°50′25.1ʺN, 102°08′01.7ʺW), from 10- to 15-cm depths, using a standard soil sampler. Soil samples were plated in serial dilutions, using sterile water, in *Streptomyces* isolation solid medium (5) supplemented with cycloheximide (100 μg/ml), nalidixic acid (30 μg/ml), and josamycin (2 μg/ml). Plates were incubated at 28°C for 5 to 7 days. *Streptomyces* sp. strain C8S0 spores were stored at –20°C in 15% glycerol. For genomic DNA extraction, 15-ml liquid cultures were inoculated with the spore suspension in ISP2 broth and incubated for 48 h at 28°C, at 200 rpm. Genomic DNA was extracted using a phenol-chloroform in-house protocol, including liquid nitrogen lysis and RNase treatment. DNA extraction was checked for quality control using a NanoDrop 1000 spectrophotometer (NanoDrop Technologies) and 1% agarose gel electrophoresis.

We sequenced the *Streptomyces* sp. strain C8S0 genome using long-read Oxford Nanopore Technologies (ONT) technology. PCR-free libraries were prepared using an ONT rapid sequencing kit (catalog number SQK-RAD003) in an R9.4 flow cell (FLO-MIN106). The sequencing cycle was run for 24 h with the built-in MinKNOW v19.06 base-calling platform Guppy v2.3.1 (ONT). Prior to read alignment and genome assembly, we ran MinIONQC, as specified elsewhere (6). Read alignment and genome assembly were performed using Flye v2.6 (7), which has been shown to be a fast and proficient tool for long-read assembly of genomes. After assembly and scaffolding, the sequence was polished using Rebaler v0.1.2 (8), using the Flye assembly as a reference. Default parameters were used for all software unless otherwise specified.

**FIG 1:**
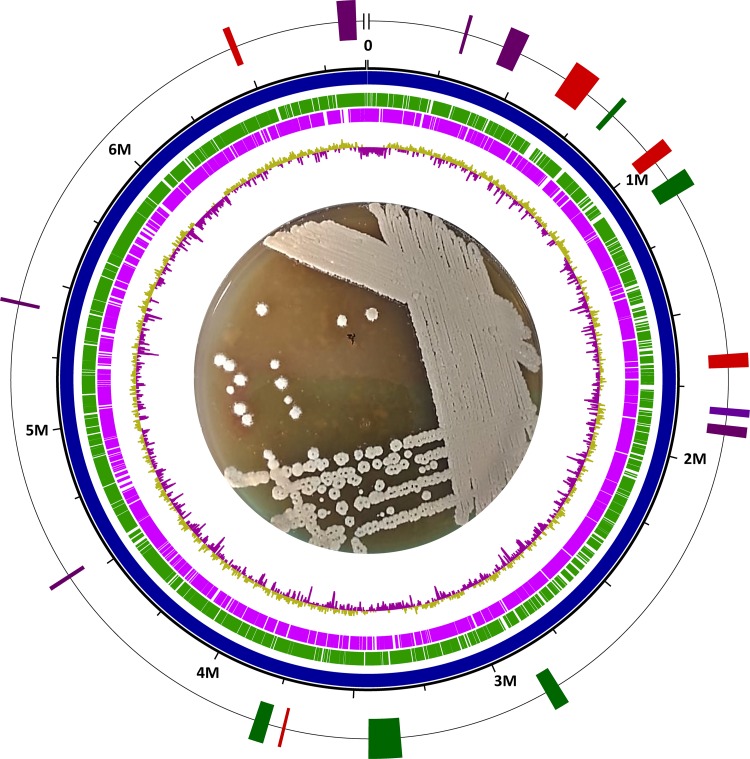
Graphical representation of the *Streptomyces* sp. C8S0 genome. From the center to the outside ring are shown GC contents (gold, above-average GC content; purple, below-average GC content), genes on the reverse strand (violet), genes on the forward strand (green), contig (blue), size marks, and BGCs with identity of >15% (green, NRPS; red, PKS; purple, terpenes; light purple, others). In the center is shown the macroscopic morphology in ISP4 medium, with yellow and brown sporulation and yellow secretions.

Using ONT MinION technology, we obtained full genomic coverage at 50× sequencing depth. The genome of *Streptomyces* sp. strain C8S0 consists of 6,898,902 bp (110 contigs; *N*_50_, 135,266 bp) assembled into a single scaffold using Flye v2.6 (7). The taxonomic identification pipeline of the Microbial Genomes Atlas (MiGA) (v0.4.1.0) (9) showed that *Streptomyces* sp. strain C8S0 shares 72.48% amino acid identity with Streptomyces glaucescens GLA.O (GenBank accession number CP009438). The Pathosystems Resource Integration Center (PATRIC) platform was used for genome annotation (10), based on the RAST tool kit (RASTtk). The genome annotation for *Streptomyces* sp. strain C8S0 includes a total of 6,841 coding sequences (CDSs), including 18 rRNA genes, 65 tRNA genes, and 68 CDSs functionally related to antibiotic resistance. In addition to phenotypic screening, we used antiSMASH v5.0 (11) as the primary source for screening the biosynthetic gene cluster (BCG) potential of *Streptomyces* sp. strain C8S0. A graphical representation of the genome and annotation is shown in Fig. 1. BGC analysis demonstrated a subset of unconventional natural product biosynthetic pathways, including nonribosomal peptide synthetase (NRPS), polyketide synthase (PKS), and terpene clusters. Further characterization of such metabolites could be relevant for finding novel activities of biomedical interest.

### Data availability.

The *Streptomyces* sp. strain C8S0 genome sequence has been deposited in DDBJ/ENA/GenBank under accession number CP045031. Raw data are available at NCBI under study PRJNA576028.
